# A simple and inexpensive method for practical storage of field-sample proteins for subsequent MALDI-TOF MS analysis

**DOI:** 10.1186/s13007-018-0358-8

**Published:** 2018-10-15

**Authors:** Michael A. Reeve, Alan G. Buddie

**Affiliations:** CABI, Bakeham Lane, Egham, Surrey TW20 9TY UK

**Keywords:** Protein extraction, Protein immobilisation, Storage stability

## Abstract

**Background:**

Protein-containing samples can readily be characterised and/or identified using matrix-assisted laser-desorption and ionisation time-of-flight mass spectrometry (MALDI-TOF MS). This technique however requires relatively-fresh biological material that contains proteins that have not yet undergone significant degradation. For field-work collection of samples, problems are often encountered due to delays between collection and sample processing, sample storage (possibly at elevated temperature and/or humidity in some climates), quarantine/regulatory restrictions on the transfer of living biological materials across national borders, and the potential to transfer unwanted microorganisms via non-living biological materials.

**Results:**

In an attempt to overcome the above difficulties, we have developed a simple and inexpensive method for practical storage of field-sample proteins, for subsequent MALDI-TOF MS analysis, in which biological material is crushed onto filter paper and dried. The dried and protein-impregnated filter paper can then be soaked in an alcoholic solution suitable for the inactivation of microorganisms of concern and again dried for storage. After subsequent dry storage, the proteins may be eluted from the paper using a solution containing acetonitrile, trifluoroacetic acid, water, and MALDI-TOF MS matrix near to saturation. The extracted proteins are then pipetted onto the MALDI-TOF MS sample plate for subsequent analysis. Using this method, spectra of comparable quality to fresh-material controls have been obtained for acid-soluble proteins from *Fallopia japonica* and *Impatiens glandulifera* leaf material. Unlike untreated leaf material, high-quality spectra can be obtained with and without alcohol treatment even after storage for one month at up to 40 °C.

**Conclusions:**

We have developed a simple and inexpensive method for practical storage of field-sample proteins for subsequent MALDI-TOF MS analysis. Key benefits of this approach are a reduction in sample degradation, and consequent conservation of taxon-discriminatory spectral profiles, whilst minimising the potential for carryover of viable microorganisms.

**Electronic supplementary material:**

The online version of this article (10.1186/s13007-018-0358-8) contains supplementary material, which is available to authorized users.

## Background

Matrix-assisted laser-desorption and ionisation time-of-flight mass spectrometry (MALDI-TOF MS) makes use of the MALDI soft ionisation process [1], in which large proteins can be prepared intact in the gas phase with predominantly a single positive charge [2]. As the time-of-flight of a charged protein, along a tube held at high vacuum, after acceleration in an electrical field, is proportional to the square root of the mass-over-charge ratio for the protein, a mass spectrum can be generated from the time-of-flight values for such gas-phase and charged protein components in a particular biological sample [3]. For the characterisation and identification of the sample, the mass spectrum of a subset of the expressed proteome is often used (normally the highly-expressed acid-soluble proteins, including many ribosomal proteins) [4].

Human clinical microbiology, particularly the diagnosis of bacterial and yeast infections, has been a key driver behind the development of MALDI-TOF MS for the identification and/or characterisation of biological samples [4]. This area has been reviewed extensively by Clark et al. [3], along with many common methods used for sample preparation. Additional methods have also been developed for mycobacteria [5], yeasts [6], and filamentous fungi [7], including the ‘full-extraction’ protocols after the work of Cassagne et al. [8]. Plant and insect materials are unfortunately not particularly well suited to many of the above methods [9] and we have, in response, developed a highly-simplified and inexpensive method for sample preparation, with broad applicability to bacteria, fungi, insects, and plants (manuscript submitted), that lyses cells by immersion (either with or without maceration) in aqueous acetonitrile containing trifluoroacetic acid (TFA) to selectively extract acid-soluble proteins, with lysis and extraction carried out in the presence of near-saturated and inexpensive-grade MALDI matrix. The resulting matrix-saturated lysate containing acid-solubilised proteins is then dried down directly onto the MALDI-TOF MS sample plate, overlaid with additional matrix solution (if required), and analysed.

Regardless of the method used for sample preparation, one limitation of MALDI-TOF MS is that it requires relatively-fresh biological material that contains proteins that have not yet undergone significant degradation. Whilst spectral profiles may be obtained from stored material, Yssouf et al. [10] showed that spectra obtained from specimens stored in 70% (v/v) ethanol were not as useful as those obtained from fresh samples for taxon discrimination in fleas. For field-work collection of samples, problems are often encountered due to: delays between collection and sample processing, sample storage (possibly at elevated temperature and/or humidity in some climates), quarantine restrictions on the transfer of living biological materials across national borders (e.g. see EC directive 2000/29/EC), and the potential to transfer microorganisms via non-living biological materials or fomites. In an attempt to overcome the above difficulties, we sought to develop a simple and inexpensive method, for the practical storage of field-sample proteins for subsequent MALDI-TOF MS analysis, in which biological material is crushed onto filter paper and dried. The dried and protein-impregnated filter paper can then be treated in order to inactivate microorganisms of concern and again dried for storage. After dry storage, the proteins are extracted from the paper using a solution containing acetonitrile, TFA, water, and MALDI-TOF MS matrix near to saturation. The extracted proteins are then pipetted onto the MALDI-TOF MS sample plate for subsequent analysis. Convenient methods for microbial inactivation include autoclaving, dry heating, and treatment with various alcohols, alone or in aqueous solution.

In the current paper, we describe the development of this method and compare the stability of stored proteins immobilised on paper with proteins obtained from untreated leaf material from *Fallopia japonica* (Japanese knotweed) and *Impatiens glandulifera* (Himalayan balsam), two plant species that were readily available to us.

## Methods

### Mass spectrometry

Mass spectrometry covering the range 2–20 kDa was carried out using a Bruker Microflex LT linear-mode instrument running the MALDI Biotyper 4.0 applications (Bruker Daltonik, Bremen, Germany), using a nitrogen laser at 337 nm, with 240 laser shots per sample, and an ion-source voltage of 19.98 kV. Calibration was carried out using the manufacturer’s ‘BTS’ controls (*E. coli* proteins supplemented with ribonuclease A and myoglobin), using peaks with masses at 3637.8; 5096.8; 5381.4; 6255.4; 7274.5; 10,300.2; 13,683.2, and 16,952.3 for calibration according to the manufacturer’s instructions. Spectra were acquired using MALDI Biotyper RTC Version 4.0 (Build 19) using the manufacturer’s standard settings (Centroid peak-detection algorithm and TopHat baseline subtraction). Database entries were made as single-spectra MSPs using the Bruker Online Client software suite (Version 4.0.19, Bruker Daltonik, Bremen, Germany) using the manufacturer’s standard settings. For spectral comparisons, Bruker identification scores were derived using the standard Bruker algorithm. This first converts raw mass spectra into peak lists, which are then compared between spectra. Three separate values are computed: the number of peaks in the reference spectrum that have a closely-matching partner in the test spectrum (value range 0–1), the number of peaks in the test spectrum that have a closely-matching partner in the reference spectrum (value range 0–1), and the peak-height symmetry of the matching peaks (value range 0–1). The above three values are multiplied together and normalised to 1000, and the base-10 logarithm is then taken to give the final Bruker score (range 0–3). Bruker scores of scores between 2.3 and 3.0 indicate very close relatedness, scores between 2.0 and 2.3 indicate close relatedness, and scores below 1.7 indicate low relatedness.

### Reagents and paper

≥ 99.8% ethanol, LC–MS-grade water, ≥ 98% (TLC-grade) α-cyano-4-hydroxycinnamic acid (HCCA) matrix, LC–MS-grade acetonitrile, 99% ReagentPlus^®^-grade TFA, and Whatman^®^ qualitative filter paper, Grade 3 (90 mm circles), were purchased from Sigma (Gillingham, UK).

### Initial time course

For MALDI-TOF MS spectra of *Fallopia japonica*-leaf and *Impatiens glandulifera*-leaf acid-soluble proteins extracted from untreated leaf material stored at 20 °C and from leaf material stored at 20 °C in 70% (v/v) ethanol, roughly 2 mm × 2 mm leaf fragments were macerated in 100 µl of (11 mg/ml HCCA matrix in 65% (v/v) acetonitrile, 2.5% (v/v) TFA, and 32.5% (v/v) water) (referred to as Solution 1 in the following) using the blunt end of a plastic inoculating loop. 1 µl of the resulting crude lysate was then pipetted onto the Bruker sample plate, air dried, and loaded into the spectrometer. For MALDI-TOF MS spectra of *Fallopia japonica*-leaf and *Impatiens glandulifera*-leaf acid-soluble proteins extracted from crushed-leaf fluids dried onto filter paper stored at 20 °C, Whatman filter paper, grade 3 was cut to roughly 5 cm × 1 cm strips. These were then covered with *Fallopia japonica* or *Impatiens glandulifera* leaf material cut to the same size. The paper was then folded to give 2.5 cm × 1 cm double-thickness leaf material enclosed in paper, which was then wrapped in cling film and gently tapped over the whole surface using a hammer against a solid surface in order to crush the leaf material and visibly stain the paper green with liquid material released from leaf cells. The cling film was then removed and the paper unfolded. Fibrous leaf remnants were removed by gently brushing with a tissue and the paper was allowed to air dry. The paper was then cut into roughly 8 mm × 2 mm strips before storage at 20 °C in Eppendorf tubes. Proteins were finally extracted by adding 100 µl of Solution 1, capping the tube, briefly vortexing, soaking for 5 min, and vortexing again. 1 µl of the supernatant was then pipetted onto the Bruker sample plate, air dried, and loaded into the spectrometer. Paper-only controls were also performed as above but with the omission of plant material. Samples were processed immediately and after storage for 3, 8, 14, 21, 30, and 36 days at 20 °C. Two plant species were used and two leaves from each species were also used. Spectral comparisons were made between the time-course sample spectra indicated and the cognate t = 0 sample using the Bruker Online Client software suite.

### Initial microbial inactivation studies

For MALDI-TOF MS spectra of *Fallopia japonica*-leaf acid-soluble proteins extracted from untreated leaf material, samples were processed as described above. For MALDI-TOF MS spectra of *Fallopia japonica*-leaf acid-soluble proteins extracted from crushed-leaf fluids dried onto filter paper and then soaked for 10 min in 70% (v/v) ethanol before re-drying, samples were processed as described above except that, after air drying, the paper was then cut into roughly 8 mm × 2 mm strips and these were immersed in 70% (v/v) ethanol for 10 min at room temperature in Eppendorf tubes. The 70% (v/v) ethanol was then removed and the paper was allowed to air dry in uncapped tubes. Proteins were finally extracted and samples were loaded into the spectrometer as described above. For MALDI-TOF MS spectra of *Fallopia japonica*-leaf acid-soluble proteins extracted from crushed-leaf fluids dried onto filter paper, samples were processed as described above, and for MALDI-TOF MS spectra of *Fallopia japonica*-leaf acid-soluble proteins extracted from crushed-leaf fluids dried onto filter paper and autoclaved before re-drying, samples were treated as described above except that the treatment with 70% (v/v) ethanol was replaced by heating at 121 °C for 30 min in an autoclave (Denley, Angus, UK). Triplicate sample preparations were carried out.

### Storage studies with *Impatiens glandulifera*-leaf acid-soluble proteins

For MALDI-TOF MS spectra of *Impatiens glandulifera***-**leaf acid-soluble proteins extracted from untreated leaf material, samples were processed as described above. For MALDI-TOF MS spectra of *Impatiens glandulifera***-**leaf acid-soluble proteins extracted from crushed-leaf fluids dried onto filter paper and then soaked for 10 min in 70% (v/v) ethanol or 70% (v/v) isopropanol before re-drying, a 90 mm-diameter Whatman filter paper, grade 3 disc was covered with *Impatiens glandulifera* leaf material cut to the same size. The paper was then folded to give a semicircle of double-thickness leaf material enclosed in paper, which was then wrapped in cling film and gently tapped over the whole surface using a hammer against a solid surface in order to crush the leaf material and visibly stain the paper green with liquid material released from leaf cells. The cling film was then removed and the paper unfolded. Fibrous leaf remnants were removed by gently brushing with a tissue and the paper was allowed to air dry. The paper was then cut into roughly 8 mm × 2 mm strips and these were immersed in either 70% (v/v) ethanol or 70% (v/v) isopropanol as indicated for 10 min at room temperature in Eppendorf tubes. The aqueous alcohol was then removed and the paper was allowed to air dry in uncapped tubes. Proteins were finally extracted as described above and 1 µl of the supernatant was then pipetted onto the Bruker sample plate, air dried, and loaded into the spectrometer. For MALDI-TOF MS spectra of *Impatiens glandulifera* leaf acid-soluble proteins extracted from crushed-leaf fluids dried onto filter paper, samples were treated as above except that the treatment with aqueous alcohol was omitted and for MALDI-TOF MS spectra of *Impatiens glandulifera* leaf acid-soluble proteins extracted from crushed-leaf fluids dried onto filter paper with dry heat treatment, samples were treated as above except that the treatment with aqueous alcohol was replaced by dry heating for 2 h at 170 °C in an oven (Gallenkamp, Loughborough, UK). Triplicate samples were analysed immediately using MALDI-TOF MS and after 35 days of storage at − 20 °C, 5 °C, 20 °C, 30 °C, and 40 °C as indicated. Spectral comparisons were made between the sample spectra indicated and the cognate replicate 1 sample processed immediately using the Bruker Online Client software suite. Average-comparison scores were calculated and error bars are shown as one standard deviation either side of the mean.

## Results

### Initial time course

**Fig. 1:**
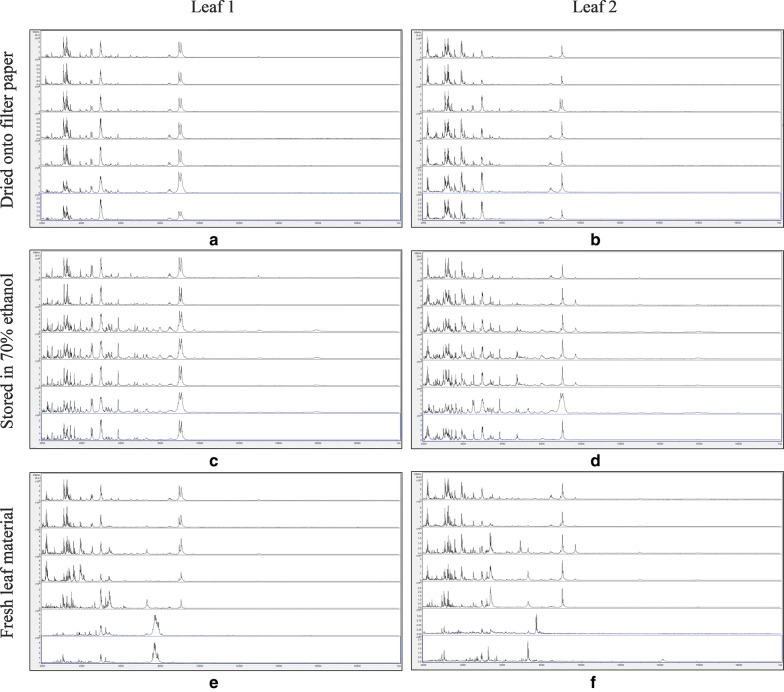
MALDI-TOF MS spectra for duplicate leaf samples of *Fallopia japonica*, with samples, from top to bottom within each panel, processed immediately and after storage for 3, 8, 14, 21, 30, and 36 days at 20 °C. The samples are: **a** leaf 1, dried down on filter paper; **b** leaf 2, dried down on filter paper; **c** leaf 1, stored in 70% (v/v) ethanol; **d** leaf 2, stored in 70% (v/v) ethanol; **e** leaf 1, untreated; and **f** leaf 2, untreated. Spectra are shown baseline-subtracted, smoothed, y-axis-autoscaled, and covering the mass range 2–20 kDa (with x-axis scale increments of 2 kDa)

Figure 1 shows MALDI-TOF MS spectra over the mass range 2–20 kDa for duplicate leaf samples of *Fallopia japonica*, dried onto filter paper, stored in 70% (v/v) ethanol, and untreated, with samples processed immediately and after storage for 3, 8, 14, 21, 30, and 36 days at 20 °C. No spectra were obtained for the filter-paper-only controls for each leaf and for each time point and these are therefore not included in Fig. 1. As can be seen from Fig. 1, spectra appeared well conserved over the time course, except for the untreated samples, where spectral deterioration was clearly evident after 30 days’ storage.

Additional file 1: Table S1 shows MALDI-TOF MS spectral comparison data for the same duplicate samples of *Fallopia japonica*, dried onto filter paper, stored in 70% (v/v) ethanol, and untreated, between samples processed immediately and samples processed immediately and after storage for 3, 8, 14, 21, 30, and 36 days at 20 °C.

**Fig. 2:**
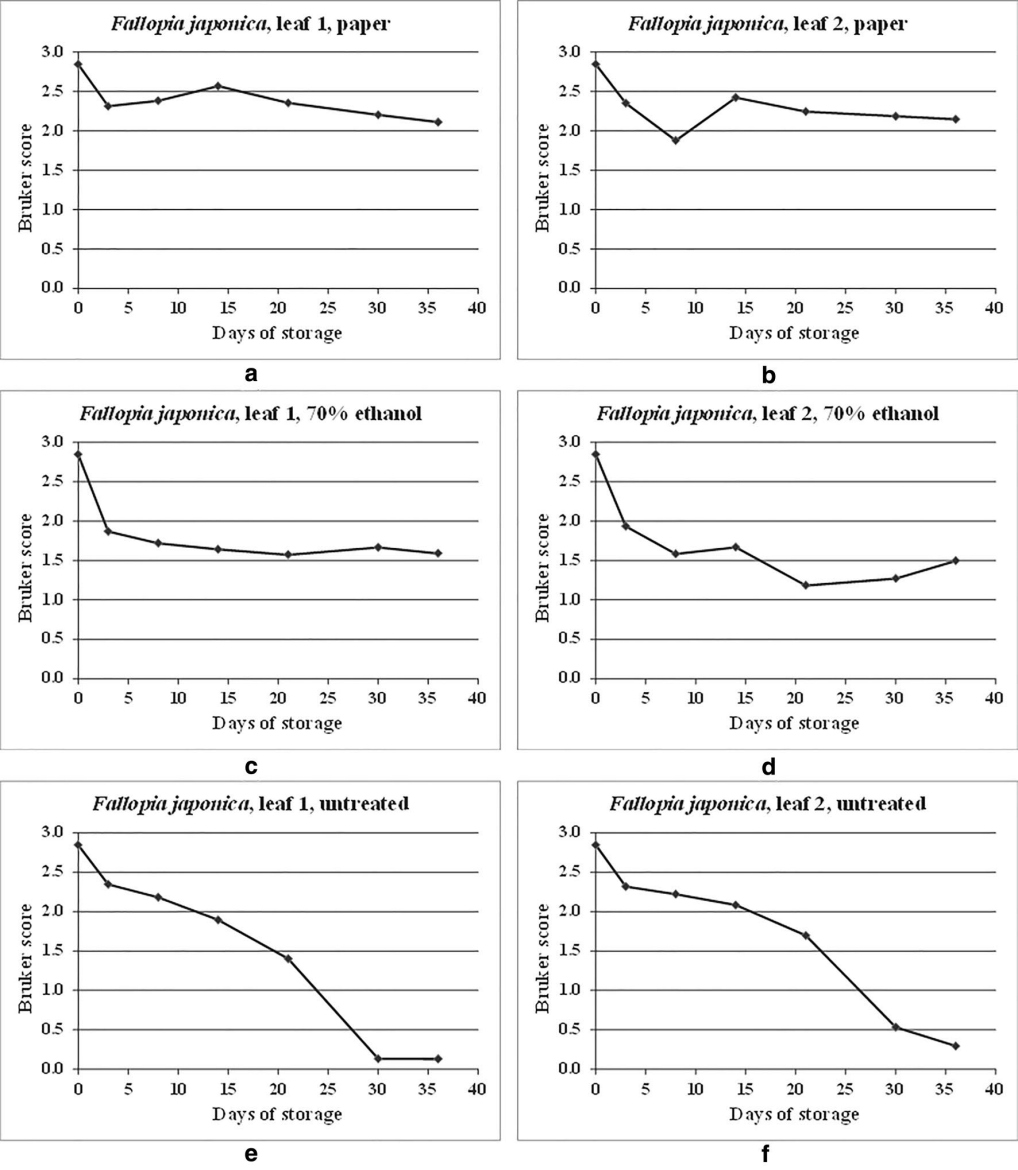
MALDI-TOF MS spectral comparisons for duplicate leaf samples of *Fallopia japonica*, dried onto filter paper, stored in 70% (v/v) ethanol, and untreated, between samples processed immediately and samples processed immediately and after storage for 3, 8, 14, 21, 30, and 36 days at 20 °C

Figure 2 shows the spectral comparison data from Additional file 1: Table S1 in graphical form. As can be seen from Additional file 1: Table S1 and Fig. 2, Bruker scores greater than 2.0 are observed for all but one time point over the time course for the samples dried onto filter paper, indicating good spectral conservation over time. For the samples stored in 70% (v/v) ethanol, Bruker scores below 2.0 are observed for all except the initial (self-against-self) reference comparison, indicating a drop in quality (as measured by spectral relatedness) after storage for just 3 days, albeit levelling off to some extent thereafter. For the untreated samples, a gradual deterioration in spectral relatedness is observed over the time course.

**Fig. 3:**
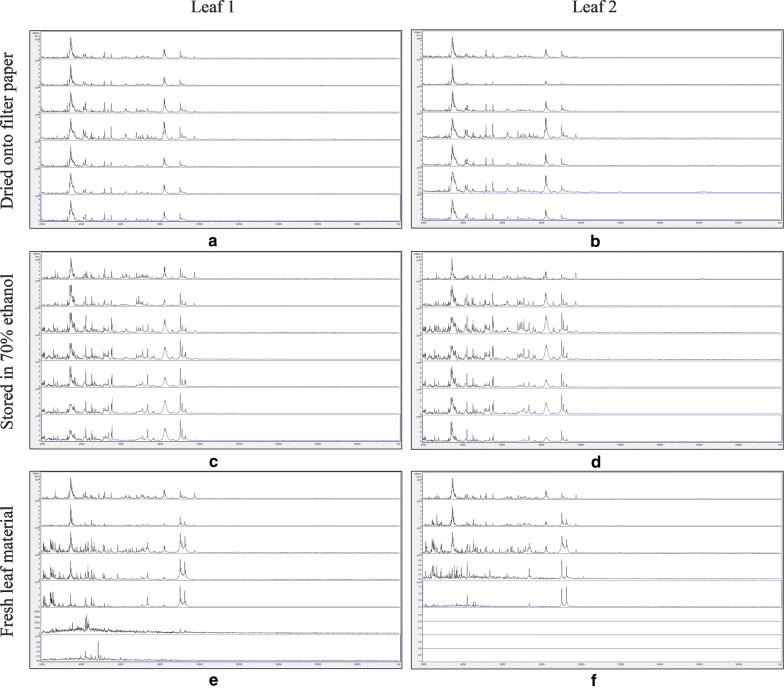
MALDI-TOF MS spectra for duplicate leaf samples of *Impatiens glandulidera*, with samples, from top to bottom within each panel, processed immediately and after storage for 3, 8, 14, 21, 30, and 36 days at 20 °C. The samples are: **a** leaf 1, dried down on filter paper; **b** leaf 2, dried down on filter paper; **c** leaf 1, stored in 70% (v/v) ethanol; **d** leaf 2, stored in 70% (v/v) ethanol; **e** leaf 1, untreated; and **f** leaf 2, untreated. Spectra are shown baseline-subtracted, smoothed, y-axis-autoscaled, and covering the mass range 2–20 kDa (with x-axis scale increments of 2 kDa)

Figure 3 shows MALDI-TOF MS spectra over the mass range 2–20 kDa for duplicate leaf samples of *Impatiens glandulifera*, dried onto filter paper, stored in 70% (v/v) ethanol, and untreated, with samples processed immediately and after storage for 3, 8, 14, 21, 30, and 36 days at 20 °C. No spectra were again obtained for the filter-paper-only controls for each leaf and for each time point and these are therefore not included in Fig. 3. As can be seen from Fig. 3, spectra appeared well conserved over the time course, except for the untreated samples, where spectral deterioration was clearly evident after 21–30 days’ storage.

Additional file 1: Table S2 shows MALDI-TOF MS spectral comparisons for the same duplicate samples of *Impatiens glandulifera*, dried onto filter paper, stored in 70% (v/v) ethanol, and untreated, between samples processed immediately and samples processed immediately and after storage for 3, 8, 14, 21, 30, and 36 days at 20 °C.

**Fig. 4:**
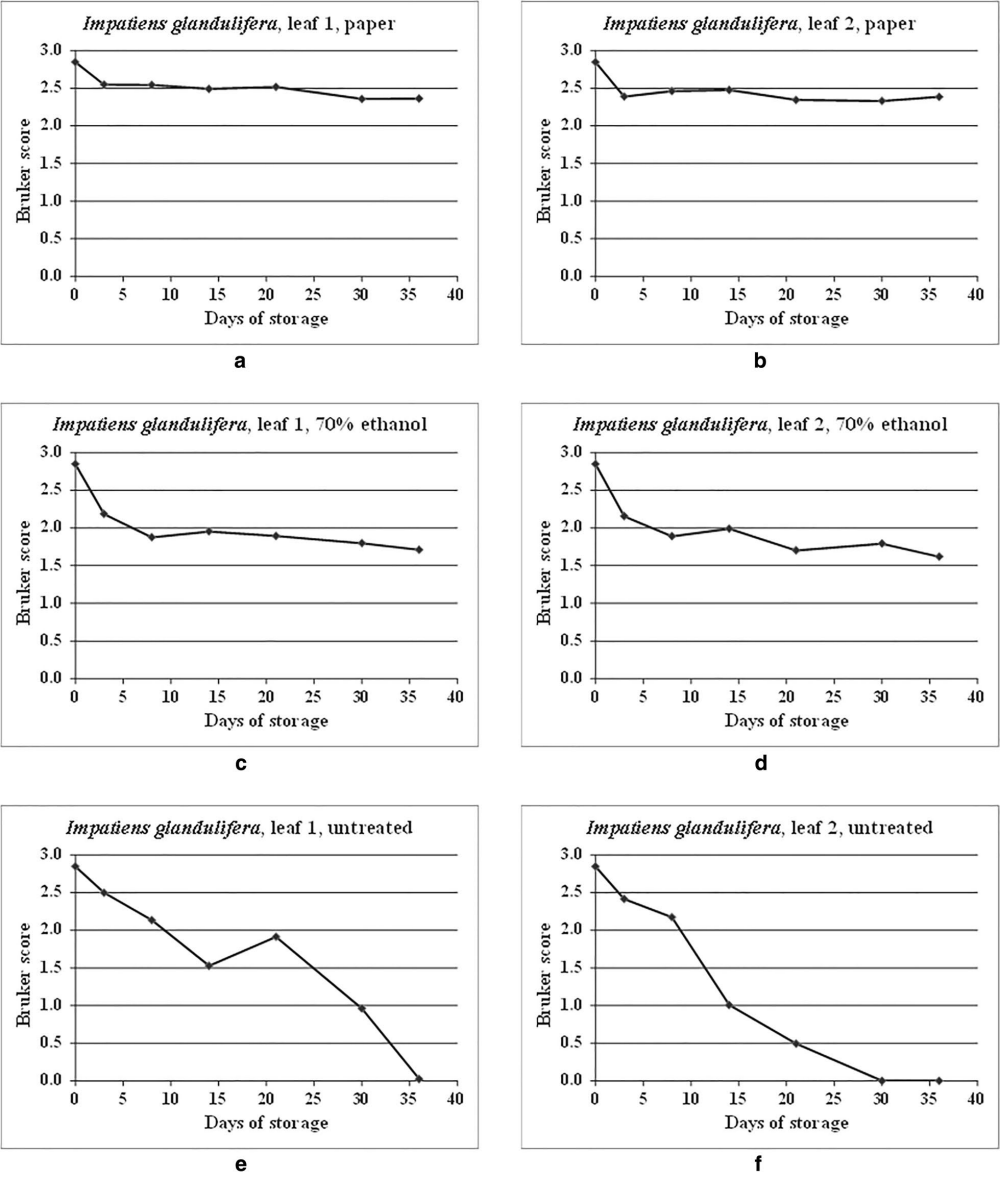
MALDI-TOF MS spectral comparisons for duplicate leaf samples of *Impatiens glandulifera*, dried onto filter paper, stored in 70% (v/v) ethanol, and untreated, between samples processed immediately and samples processed immediately and after storage for 3, 8, 14, 21, 30, and 36 days at 20 °C

Figure 4 shows the spectral comparison data from Additional file 1: Table S2 in graphical form. As can be seen from Additional file 1: Table S2 and Fig. 4, Bruker scores greater than 2.0 were observed for all time points over the time course for the samples dried onto filter paper, indicating good spectral conservation over time. For the samples stored in 70% (v/v) ethanol, Bruker scores below 2.0 were observed for all except the initial (self-against-self) reference comparison and the time point at 3 days, indicating a drop in spectral relatedness after storage for 8 days, albeit once more levelling off to some extent thereafter. For the untreated samples, a gradual deterioration in spectral relatedness was again observed over the time course.

Having demonstrated stable storage of plant-cell proteins dried down onto paper, we then investigated whether the method could be adapted to include a microbial-inactivation step, for which treatment with 70% (v/v) ethanol and autoclaving were initially assessed.

**Fig. 5:**
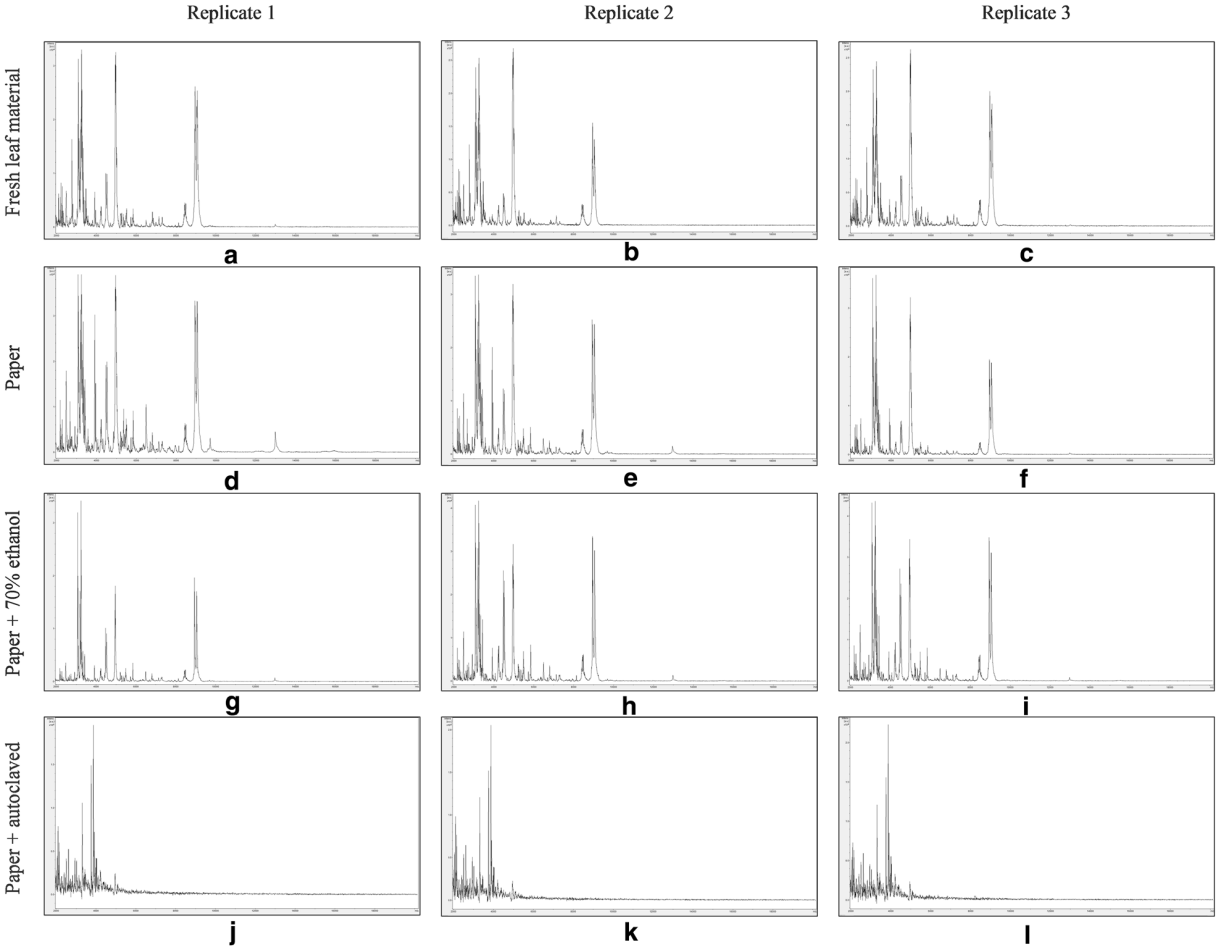
MALDI-TOF MS spectra of *Fallopia japonica*-leaf acid-soluble proteins: extracted from fresh leaf material (**a**–**c**), extracted from crushed-leaf fluids dried onto filter paper (**d**–**f**), extracted from crushed-leaf fluids dried onto filter paper and then soaked for 10 min in 70% (v/v) ethanol before re-drying (**g**–**i**), and extracted from crushed-leaf fluids dried onto filter paper and then autoclaved before re-drying (**j**–**l**). Spectra are shown baseline-subtracted, smoothed, y-axis-autoscaled, and covering the mass range 2–20 kDa (with x-axis scale increments of 2 kDa)

Figure 5 shows MALDI-TOF MS spectra over the mass range 2 kDa to 20 kDa from triplicate sample preparations of *Fallopia japonica*-leaf acid-soluble proteins extracted from: fresh leaf material (Fig. 5a–c), crushed-leaf fluids dried onto filter paper (Fig. 5d–f), crushed-leaf fluids dried onto filter paper and then soaked for 10 min in 70% (v/v) ethanol before re-drying (Fig. 5g–i), and crushed-leaf fluids dried onto filter paper and autoclaved before re-drying (Fig. 5j–l).

Whilst the spectra for the samples extracted from crushed-leaf fluids dried onto filter paper and autoclaved before re-drying (Fig. 5j–l) displayed extensive protein degradation, the spectra for the remaining samples showed good reproducibility between replicate sample preparations and good conservation of spectral profiles. Comparable spectral profiles were observed between the samples extracted from fresh leaf material and the samples extracted from crushed-leaf fluids dried onto filter paper (with and without soaking in 70% (v/v) ethanol before re-drying), with only sample (g) showing a slight diminution in peak richness. *Fallopia japonica*-leaf acid-soluble-protein MALDI-TOF MS spectra comparable to those obtained from fresh leaf material can thus be obtained using a method in which the proteins have been dried down into a solid matrix (which should inhibit degradation by proteases released from lysed cells, thereby potentially facilitating extended storage under dry conditions) and in which the dried-down material has been treated with the commonly-used microbial inactivant 70% (v/v) ethanol.

Having successfully adapted the method to include treatment with 70% (v/v) ethanol, we then investigated the influence of temperature on sample storage, also including treatment with 70% (v/v) isopropanol and dry heating as additional methods for microbial inactivation.

### Storage studies with *Impatiens glandulifera*-leaf acid-soluble proteins

**Fig. 6:**
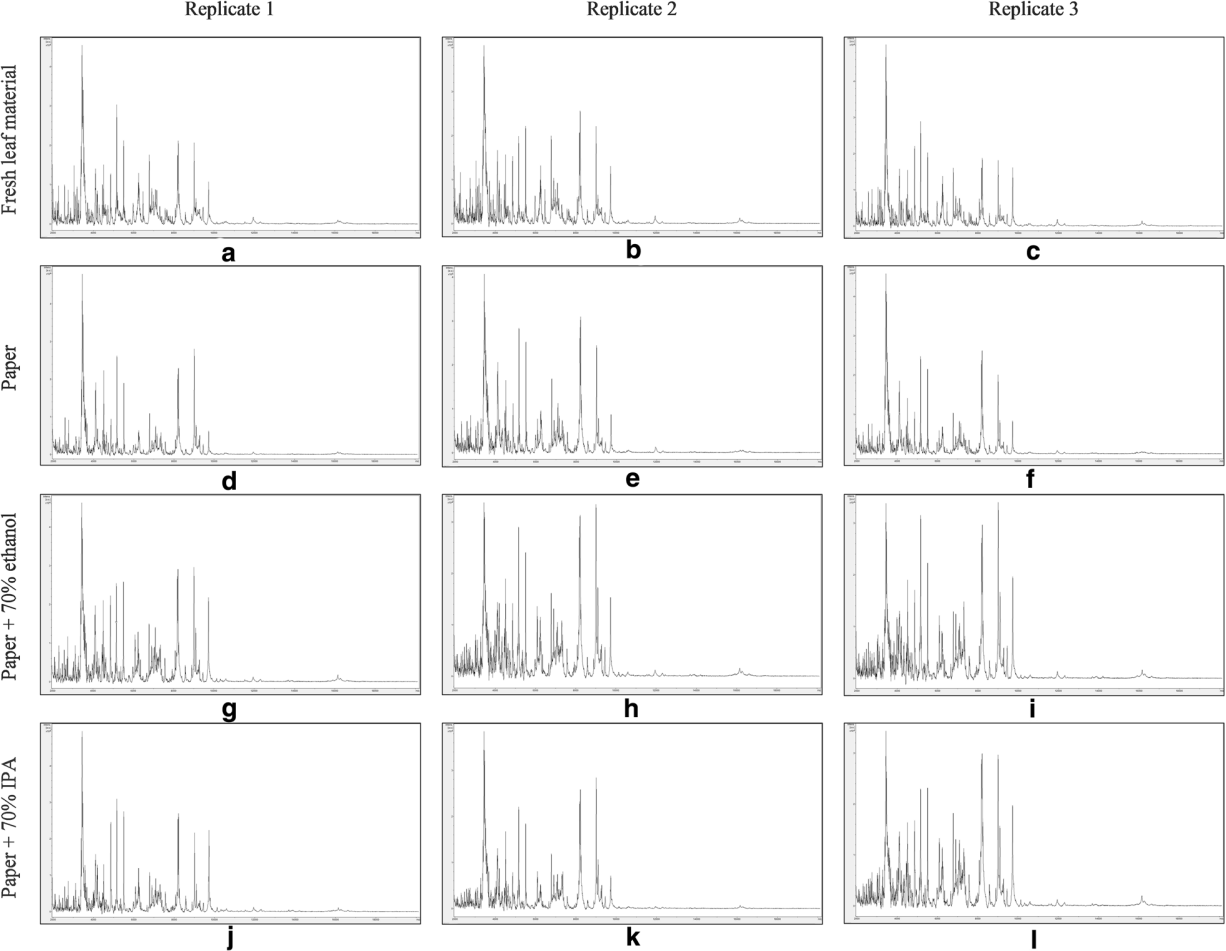
t = 0 MALDI-TOF MS spectra of *Impatiens glandulifera*-leaf acid-soluble proteins: extracted from fresh leaf material (**a**–**c**), extracted from crushed-leaf fluids dried onto filter paper (**d**–**f**), extracted from crushed-leaf fluids dried onto filter paper and then soaked for 10 min in 70% (v/v) ethanol before re-drying (**g**–**i**), and extracted from crushed-leaf fluids dried onto filter paper and then soaked for 10 min in 70% (v/v) isopropanol before re-drying (**j**–**l**). Spectra are shown baseline-subtracted, smoothed, y-axis-autoscaled, and covering the mass range 2–20 kDa (with x-axis scale increments of 2 kDa)

Figure 6 shows t = 0 MALDI-TOF MS spectra over the mass range 2–20 kDa from triplicate sample preparations of *Impatiens glandulifera***-**leaf acid-soluble proteins extracted from: fresh leaf material (Fig. 6a–c), crushed-leaf fluids dried onto filter paper (Fig. 6d–f), crushed-leaf fluids dried onto filter paper and then soaked for 10 min in 70% (v/v) ethanol before re-drying (Fig. 6g–i), and crushed-leaf fluids dried onto filter paper and then soaked for 10 min in 70% (v/v) isopropanol before re-drying (Fig. 6j–l).

No spectra were obtained for samples extracted from crushed-leaf fluids dried onto filter paper and heated for 2 h at 170 °C (data not shown, and samples not subjected to further analysis). The spectra for the remaining samples however showed good reproducibility between replicate sample preparations and good conservation of spectral profiles between the samples extracted from fresh leaf material and the samples extracted from crushed-leaf fluids dried onto filter paper with and without soaking in 70% (v/v) ethanol or 70% (v/v) isopropanol before re-drying. *Impatiens glandulifera***-**leaf acid-soluble-protein MALDI-TOF MS spectra comparable to those obtained from fresh leaf material can thus be obtained using a method in which the proteins have been dried down into a solid matrix and in which the dried-down material has been treated with the commonly-used microbial inactivants 70% (v/v) ethanol and 70% (v/v) isopropanol.

**Fig. 7:**
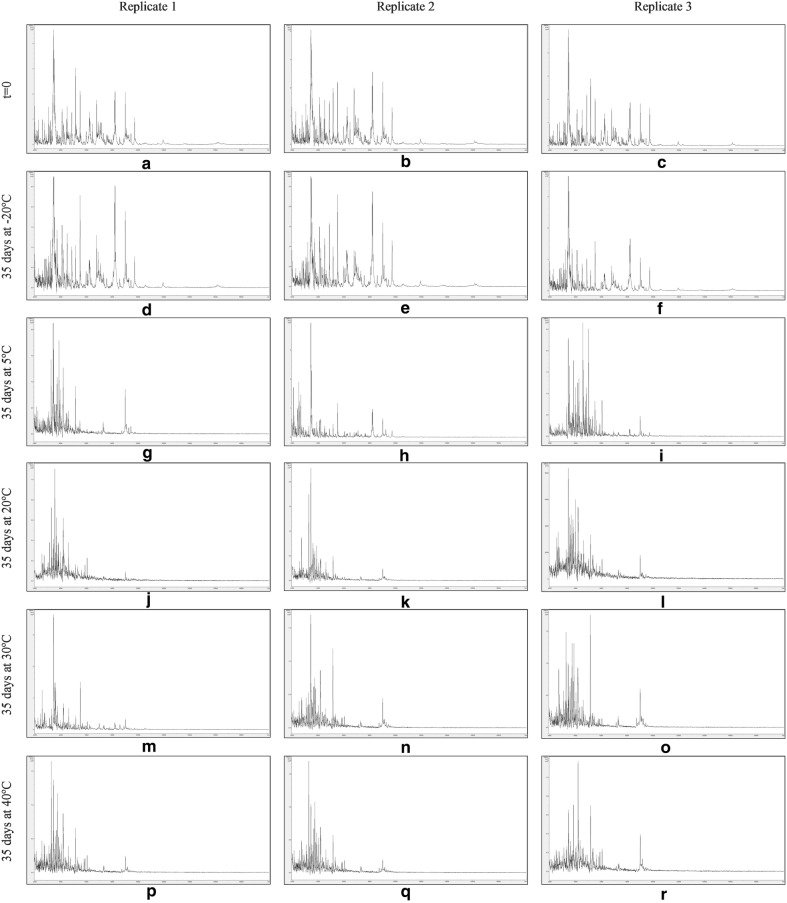
MALDI-TOF MS spectra from triplicate sample preparations of *Impatiens glandulifera*-leaf acid-soluble proteins extracted from fresh leaf material at t = 0 (**a**–**c**), and untreated leaf material after storage for 35 days at − 20 °C (**d**–**f**), 5 °C (**g**–**i**), 20 °C (**j**–**l**), 30 °C (**m**–**o**), and 40 °C (**p**–**r**). Spectra are shown baseline-subtracted, smoothed, y-axis-autoscaled, and covering the mass range 2–20 kDa (with x-axis scale increments of 2 kDa)

**Fig. 8:**
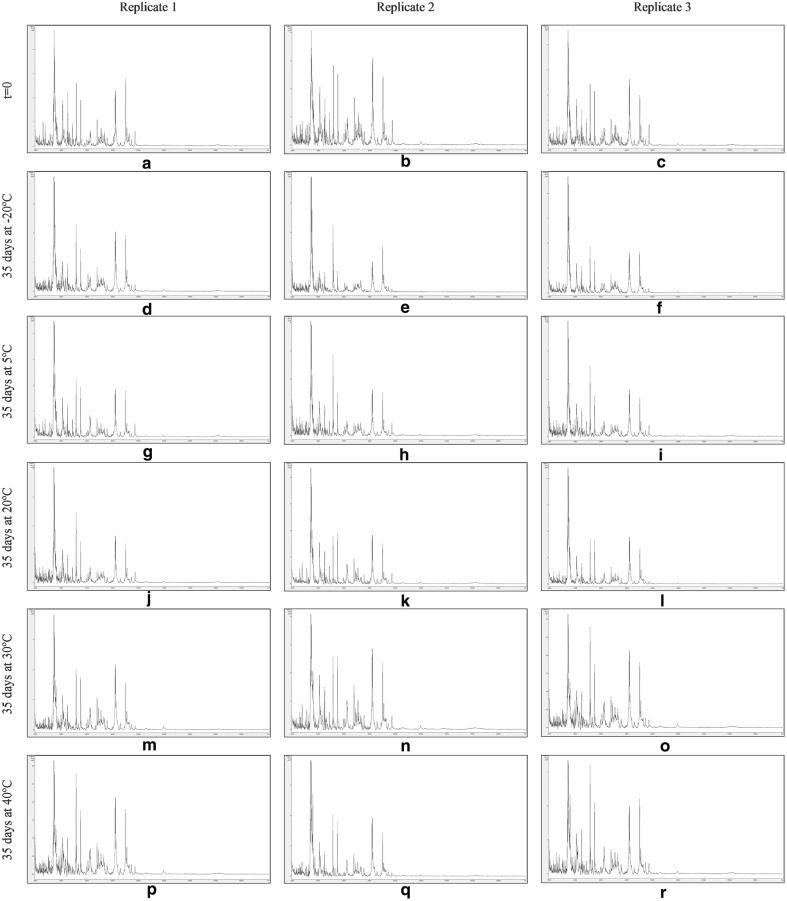
MALDI-TOF MS spectra from triplicate sample preparations of *Impatiens glandulifera*-leaf acid-soluble proteins extracted from crushed-leaf fluids dried onto filter paper at t = 0 (**a**–**c**), and after storage for 35 days at − 20 °C (**d**–**f**), 5 °C (**g**–**i**), 20 °C (**j**–**l**), 30 °C (**m**–**o**), and 40 °C (**p**–**r**). Spectra are shown baseline-subtracted, smoothed, y-axis-autoscaled, and covering the mass range 2–20 kDa (with x-axis scale increments of 2 kDa)

**Fig. 9:**
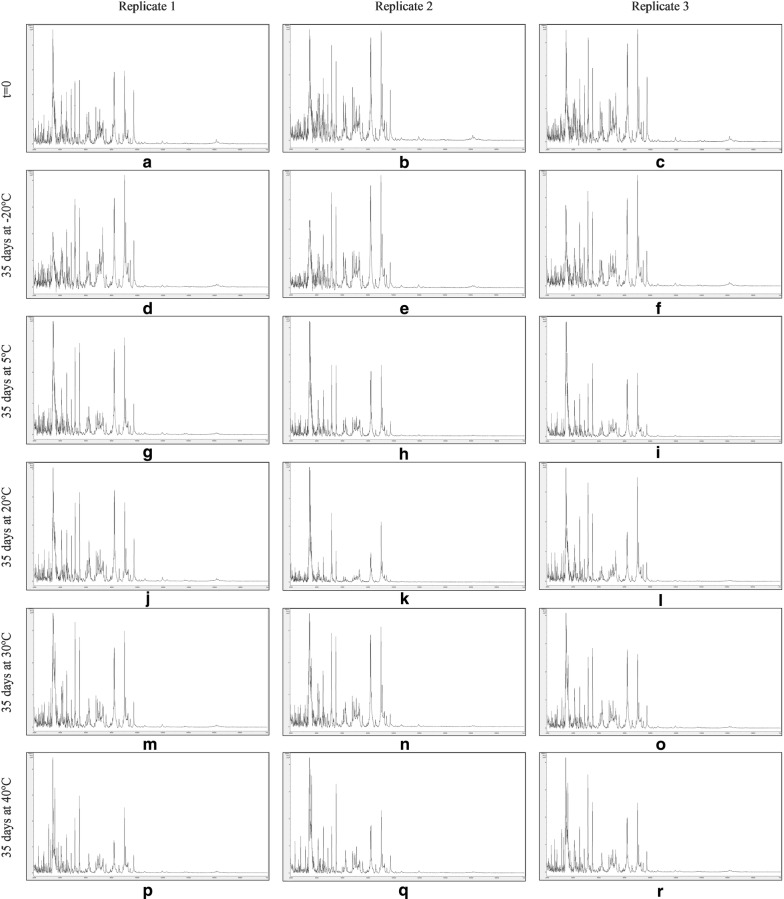
MALDI-TOF MS spectra from triplicate sample preparations of *Impatiens glandulifera*-leaf acid-soluble proteins extracted from crushed-leaf fluids dried onto filter paper with soaking for 10 min in 70% (v/v) ethanol before re-drying at t = 0 (**a**–**c**), and after storage for 35 days at − 20 °C (**d**–**f**), 5 °C (**g**–**i**), 20 °C (**j**–**l**), 30 °C (**m**–**o**), and 40 °C (**p**–**r**). Spectra are shown baseline-subtracted, smoothed, y-axis-autoscaled, and covering the mass range 2–20 kDa (with x-axis scale increments of 2 kDa)

**Fig. 10:**
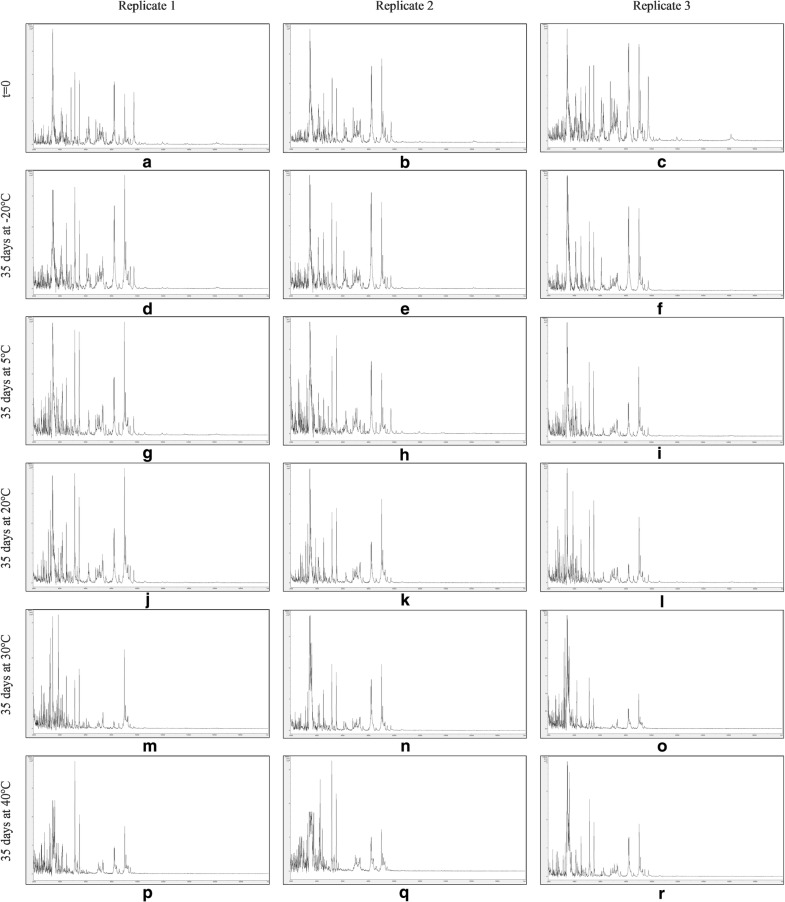
MALDI-TOF MS spectra from triplicate sample preparations of *Impatiens glandulifera*-leaf acid-soluble proteins extracted from crushed-leaf fluids dried onto filter paper with soaking for 10 min in 70% (v/v) isopropanol before re-drying at t = 0 (**a**–**c**), and after storage for 35 days at − 20 °C (**d**–**f**), 5 °C (**g**–**i**), 20 °C (**j**–**l**), 30 °C (**m**–**o**), and 40 °C (**p**–**r**). Spectra are shown baseline-subtracted, smoothed, y-axis-autoscaled, and covering the mass range 2–20 kDa (with x-axis scale increments of 2 kDa)

Figures 7, 8, 9 and 10 show MALDI-TOF MS spectra over the mass range 2–20 kDa from triplicate sample preparations of *Impatiens glandulifera***-**leaf acid-soluble proteins extracted at t = 0 (a–c) and after storage for 35 days at − 20 °C (d–f), 5 °C (g–i), 20 °C (j–l), 30 °C (m–o), and 40 °C (p–r) from untreated leaf material (Fig. 7), crushed-leaf fluids dried onto filter paper (Fig. 8), crushed-leaf fluids dried onto filter paper with soaking for 10 min in 70% (v/v) ethanol before re-drying (Fig. 9), and crushed-leaf fluids dried onto filter paper with soaking for 10 min in 70% (v/v) isopropanol before re-drying (Fig. 10).

The spectra in Fig. 7 show that, after one month of storage at between − 20 and 40 °C, there was considerable variation in spectral quality compared to t = 0 controls for *Impatiens glandulifera***-**leaf acid-soluble proteins extracted from excised leaf material, with a marked deterioration in spectra for all storage conditions except freezing at − 20 °C. The spectra in Figs. 8, 9 and 10 demonstrated that, after one month of storage at between − 20 °C and 40 °C, spectra of comparable quality to t = 0 controls were obtained for *Impatiens glandulifera***-**leaf acid-soluble proteins extracted from crushed-leaf fluids dried onto filter paper, with and without alcohol treatment.

Additional file 1: Tables S3–S6 show Bruker scores for spectral comparisons between t = 0 replicate 1 and cognate samples processed immediately and after storage for 35 days at − 20 °C, 5 °C, 20 °C, 30 °C, and 40 °C for acid-soluble proteins extracted from untreated samples (Additional file 1: Table S3); crushed-leaf fluids dried onto filter paper (Additional file 1: Table S4); crushed-leaf fluids dried onto filter paper with soaking for 10 min in 70% (v/v) ethanol before re-drying (Additional file 1: Table S5); and crushed-leaf fluids dried onto filter paper with soaking for 10 min in 70% (v/v) isopropanol before re-drying (Additional file 1: Table S6).

**Fig. 11:**
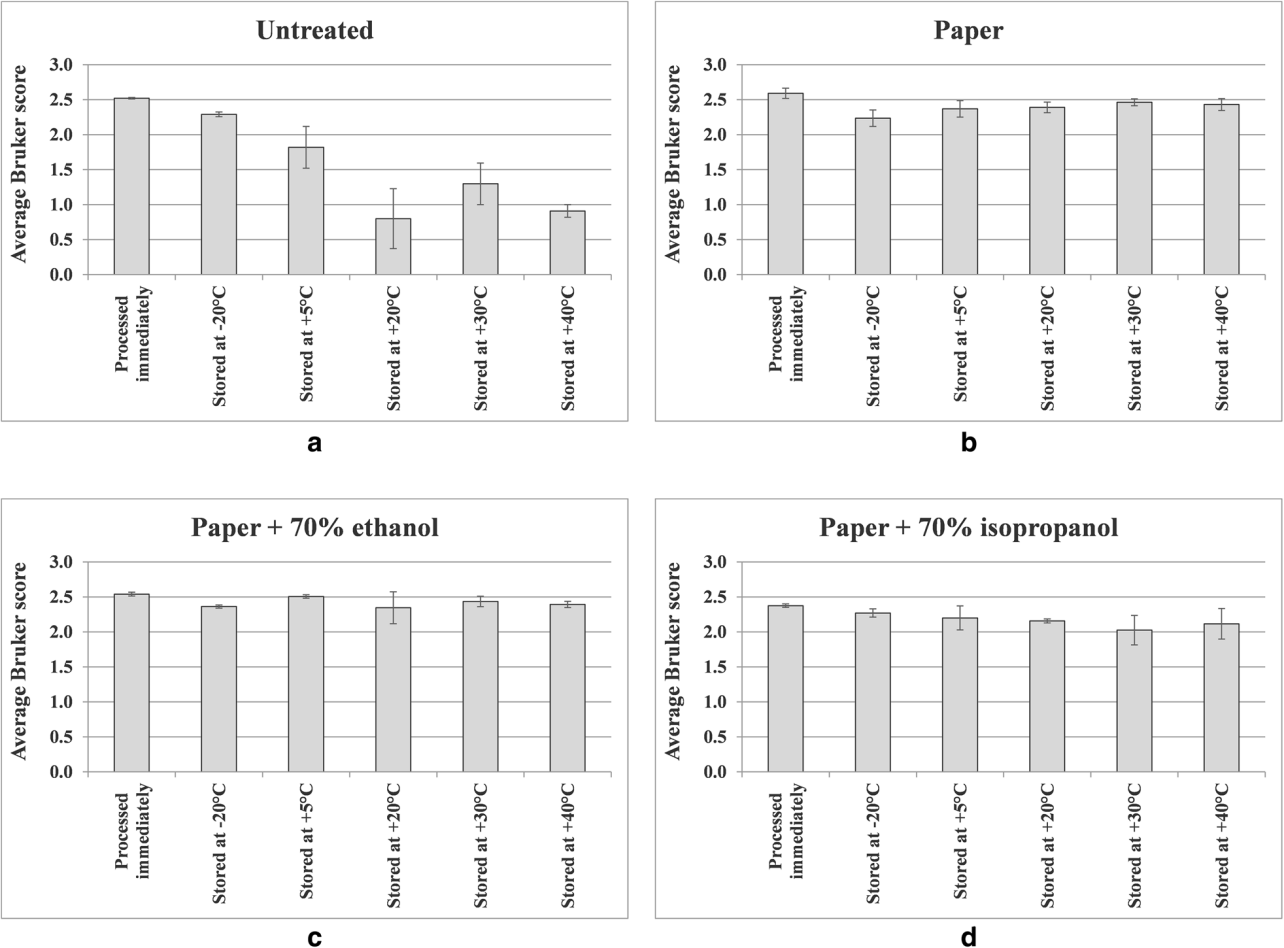
MALDI-TOF MS spectral comparisons for triplicate leaf samples of *Impatiens glandulifera*, processed immediately and after storage for 35 days at − 20 °C, 5 °C, 20 °C, 30 °C, and 40 °C as indicated, for **a** untreated samples, **b** acid-soluble proteins extracted from crushed-leaf fluids dried onto filter paper, **c** acid-soluble proteins extracted from crushed-leaf fluids dried onto filter paper with soaking for 10 min in 70% (v/v) ethanol before re-drying, and **d** acid-soluble proteins extracted from crushed-leaf fluids dried onto filter paper with soaking for 10 min in 70% (v/v) isopropanol before re-drying

Figure 11 shows MALDI-TOF MS average Bruker scores from Additional file 1: Tables S3–S6, with errors bars indicating one standard deviation either side of the mean, for triplicate leaf samples of *Impatiens glandulifera*, processed immediately and after storage for 35 days at − 20 °C, 5 °C, 20 °C, 30 °C, and 40 °C as indicated, for untreated samples; acid-soluble proteins extracted from crushed-leaf fluids dried onto filter paper; acid-soluble proteins extracted from crushed-leaf fluids dried onto filter paper with soaking for 10 min in 70% (v/v) ethanol before re-drying; and acid-soluble proteins extracted from crushed-leaf fluids dried onto filter paper with soaking for 10 min in 70% (v/v) isopropanol before re-drying.

As can be seen from Figs. 7, 8, 9, 10 and 11 and Additional file 1: Tables S3–S6, average Bruker scores for untreated samples fell below 2.0 for all but frozen storage for 35 days at − 20 °C. For dried storage on paper, with and without alcohol treatment, average Bruker scores above 2.0 were obtained for all storage conditions, albeit with a noticeable reduction in spectral conservation when 70% (v/v) isopropanol was used instead of 70% (v/v) ethanol as the microbial inactivant. Good spectral conservation is therefore possible for extended storage commensurate with field-work study across a wide range of temperatures for protein samples dried onto paper with and without microbial-inactivant treatment, for which 70% (v/v) ethanol slightly outperformed 70% (v/v) isopropanol.

## Discussion

MALDI-TOF MS is a versatile and powerful tool for the characterisation and identification of protein-containing samples, often through the analysis of their highly-expressed acid-soluble sub-proteome. Driven to a large extent by human clinical microbiology, numerous methods have been developed for sample preparation, and others are being developed for a wider range of biological materials. Notwithstanding past and current developments in sample preparation however, MALDI-TOF MS has a requirement for relatively-fresh biological material, containing proteins that have not yet undergone significant degradation. In many situations, such fresh samples are readily available but for field-work collection of samples, particularly in remote locations, problems are often encountered due to: delays between collection and sample processing, extended sample storage (possibly at elevated temperature and/or humidity in some climates), quarantine restrictions on the transfer of living biological materials across national borders, and the potential to transfer microorganisms via non-living biological materials. In an attempt to overcome the above difficulties, we sought to develop a simple and inexpensive method for the practical storage of field-sample proteins for subsequent MALDI-TOF MS analysis.

In developing our method, we reasoned that the predominant mechanism for protein degradation is likely to be contact between the above highly-expressed acid-soluble proteins and proteases released from subcellular compartments after death of the biological specimen; a process that would be favoured by extended storage at elevated temperatures up to the optimal temperature for the proteases. Drying cellular contents into a solid support would effectively separate the highly-expressed acid-soluble proteins and proteases by immobilising these onto the support. For these studies using plant-leaf material, we chose to use simple and readily accessible filter paper with high absorptive capacity (Whatman, grade 3). For future studies, we intend to investigate other solid supports but, given that we are aiming for simple immobilisation by drying, the expectation is that the nature of the support is unlikely to be critical provided that it has a reasonable absorptive capacity and that it is not adversely affected by immersion in acetonitrile, TFA, and matrix.

For microbial inactivation, we chose to test dry heat treatment (2 h at 170 °C), autoclaving (30 min at 121 °C), immersion in 70% (v/v) ethanol, and immersion in 70% (v/v) isopropanol. These alcohol-based disinfectants were tested since, although they only act on vegetative cells, they do have broad-spectrum antimicrobial activity [11], and are commonly employed in laboratories for microbial inactivation.

For extraction of proteins from the dried paper we chose to use acetonitrile containing TFA to selectively extract acid-soluble proteins, with extraction carried out in the presence of near-saturated and inexpensive-grade MALDI matrix. The resulting matrix-saturated lysate containing acid-solubilised proteins can then simply be dried down directly onto the MALDI-TOF MS sample plate and analysed. TFA was chosen for acidification because it is a significantly stronger acid than formic acid, which is frequently used in MALDI-TOF MS sample-preparation methods [8]. Using TFA therefore allows comparable proton concentrations to be obtained from much lower concentrations of acid, thereby significantly reducing the amount of odorous material evaporating from the reagent and sample plate during use. In addition, many methods described to date (reviewed in [3]) extract protein, which is then dried down onto the sample plate prior to overlay with matrix solution. As some proteins, once dried down onto solid surfaces, can be difficult to re-solubilise, we chose to premix the extracted proteins and matrix and to dry these down together, using inexpensive matrix in order to make this economically practical (in these studies, we used C2020-10G HCCA matrix supplied by Sigma, which is around 500 times cheaper per gram than many 99%-pure ‘MALDI-grade’ reagents). Whilst Niare et al. [12] have successfully used MALDI-TOF MS for the identification of *Anopheles gambiae* Giles blood meal crushed on Whatman filter papers, our method is, to the best of our knowledge, the first application of filter-paper storage and subsequent MALDI-TOF MS analysis to plant material and, compared to the method of Niare et al., uses a simpler (single-step) extraction process, with the additional option of a microbial inactivation step.

Throughout these studies, we have been able to obtain mass spectra that contain numerous peaks and which are not overwhelmed by one or a small number of dominant protein peaks. This is reassuring given previous comments reported by Mehta and Silva [9] concerning potential problems resulting from very high abundance proteins such as ribulose bisphosphate carboxylase-oxidase (RuBisCO) that are associated with photosynthesis in plant tissues. As our method selectively extracts only the acid-soluble fraction of proteins that are also soluble in 65% (v/v) acetonitrile, we speculate that the reason for this is that RUBISCO and other potentially-problematic very high abundance proteins are not extracted from the filter papers under these conditions.

The observed spectra for samples dried down onto filter paper and then extracted (with and without prior alcohol treatment) are slightly less complex than those observed from fresh leaf material and from leaf material stored under 70% (v/v) ethanol. Whilst this might mean that a small number of proteins could be excluded from the MALDI-TOF MS spectra obtained with our method, we believe that the ability to carry out simple, inexpensive, and reproducible MALDI-TOF MS-based analysis of plant material without recourse to freezing immediately after sampling and keeping samples constantly frozen all the way from field-work sites to the laboratory more than compensates for this.

Having demonstrated good spectral conservation for extended storage commensurate with field-work study across a wide range of temperatures for protein samples dried onto paper with and without microbial-inactivant treatment, we plan in future studies to test this method across further plant taxa.

## Conclusions

We have developed a simple and inexpensive method for practical storage of field-sample proteins, for subsequent MALDI-TOF MS analysis, in which biological material is crushed onto filter paper and dried. The dried and protein-impregnated filter paper can be soaked in an alcoholic solution suitable for the inactivation of microorganisms of concern and can again be dried for storage. After dry storage, proteins can be eluted from the paper using a solution containing acetonitrile, TFA, water, and MALDI-TOF MS matrix near to saturation. Using this method, spectra of comparable quality to fresh-material controls can be obtained for acid-soluble proteins from *Fallopia japonica* and *Impatiens glandulifera* leaf material. Unlike untreated leaf material, high-quality spectra can be obtained with and without alcohol treatment even after storage for one month at up to 40 °C. Key benefits of this approach are a reduction in sample degradation (thereby maintaining field-obtained sample integrity), and consequent conservation of taxon-discriminatory spectral profiles, whilst minimising the potential for carryover of viable microorganisms.

## Additional file

**Additional file 1: Table S1.** shows MALDI-TOF MS spectral comparison data for the same duplicate samples of *Fallopia japonica*, dried onto filter paper, stored in 70% (v/v) ethanol, and untreated, between samples processed immediately and samples processed immediately and after storage for 3, 8, 14, 21, 30, and 36 days at 20 °C. **Table S2.** shows MALDI-TOF MS spectral comparisons for duplicate samples of *Impatiens glandulifera*, dried onto filter paper, stored in 70% (v/v) ethanol, and untreated, between samples processed immediately and samples processed immediately and after storage for 3, 8, 14, 21, 30, and 36 days at 20 °C. **Tables S3–S6** show Bruker scores for *Impatiens glandulifera*-leaf acid-soluble-protein spectral comparisons between t = 0 replicate 1 and cognate samples processed immediately and after storage for 35 days at − 20 °C, 5 °C, 20 °C, 30 °C, and 40 °C for acid-soluble proteins extracted from untreated samples (**Table S3**); crushed-leaf fluids dried onto filter paper (**Table S4**); crushed-leaf fluids dried onto filter paper with soaking for 10 min in 70% (v/v) ethanol before re-drying (**Table S5**); and crushed-leaf fluids dried onto filter paper with soaking for 10 min in 70% (v/v) isopropanol before re-drying (**Table S6**).

## Abbreviations

MALDI-TOF MS: matrix-assisted laser-desorption and ionisation time-of-flight mass spectrometry
HCCA: α-cyano-4-hydroxycinnamic acid
TFA: trifluoroacetic acid
