# Supplementary material for: A simple and inexpensive method for practical storage of field-sample proteins for subsequent MALDI-TOF MS analysis
Source: Plant Methods. 2018 Oct 15;14:90. doi: 10.1186/s13007-018-0358-8 (PMC6192001; doi:10.1186/s13007-018-0358-8)
Supplement: Supplementary file 1 — Additional file 1: Table S1. shows MALDI-TOF MS spectral comparison data for the same duplicate samples of Fallopia japonica, dried onto filter paper, stored in 70% (v/v) ethanol, and untreated, between samples processed immediately and samples processed immediately and after storage for 3, 8, 14, 21, 30, and 36 days at 20 °C. Table S2. shows MALDI-TOF MS spectral comparisons for duplicate samples of Impatiens glandulifera, dried onto filter paper, stored in 70% (v/v) ethanol, and untreated, between samples processed immediately and samples processed immediately and after storage for 3, 8, 14, 21, 30, and 36 days at 20 °C. Tables S3–S6 show Bruker scores for Impatiens glandulifera-leaf acid-soluble-protein spectral comparisons between t = 0 replicate 1 and cognate samples processed immediately and after storage for 35 days at − 20 °C, 5 °C, 20 °C, 30 °C, and 40 °C for acid-soluble proteins extracted from untreated samples (Table S3); crushed-leaf fluids dried onto filter paper (Table S4); crushed-leaf fluids dried onto filter paper with soaking for 10 min in 70% (v/v) ethanol before re-drying (Table S5); and crushed-leaf fluids dried onto filter paper with soaking for 10 min in 70% (v/v) isopropanol before re-drying (Table S6). [file 13007_2018_358_MOESM1_ESM.docx]

**Table S1**

| Reference spectrum | Comparison spectrum | Bruker score |
| --- | --- | --- |
| *Fallopia japonica*, leaf 1, paper, t=0 days | *Fallopia japonica*, leaf 1, paper, t=0 days | 2.845 |
|  | *Fallopia japonica*, leaf 1, paper, t=3 days | 2.314 |
|  | *Fallopia japonica*, leaf 1, paper, t=8 days | 2.382 |
|  | *Fallopia japonica*, leaf 1, paper, t=14 days | 2.569 |
|  | *Fallopia japonica*, leaf 1, paper, t=21 days | 2.355 |
|  | *Fallopia japonica*, leaf 1, paper, t=30 days | 2.204 |
|  | *Fallopia japonica*, leaf 1, paper, t=36 days | 2.111 |
| *Fallopia japonica*, leaf 1, 70% ethanol, t=0 days | *Fallopia japonica*, leaf 1, 70% ethanol, t=0 days | 2.845 |
|  | *Fallopia japonica*, leaf 1, 70% ethanol, t=3 days | 1.868 |
|  | *Fallopia japonica*, leaf 1, 70% ethanol, t=8 days | 1.719 |
|  | *Fallopia japonica*, leaf 1, 70% ethanol, t=14 days | 1.641 |
|  | *Fallopia japonica*, leaf 1, 70% ethanol, t=21 days | 1.573 |
|  | *Fallopia japonica*, leaf 1, 70% ethanol, t=30 days | 1.666 |
|  | *Fallopia japonica*, leaf 1, 70% ethanol, t=36 days | 1.590 |
| *Fallopia japonica*, leaf 1, untreated, t=0 days | *Fallopia japonica*, leaf 1, untreated, t=0 days | 2.845 |
|  | *Fallopia japonica*, leaf 1, untreated, t=3 days | 2.347 |
|  | *Fallopia japonica*, leaf 1, untreated, t=8 days | 2.182 |
|  | *Fallopia japonica*, leaf 1, untreated, t=14 days | 1.894 |
|  | *Fallopia japonica*, leaf 1, untreated, t=21 days | 1.401 |
|  | *Fallopia japonica*, leaf 1, untreated, t=30 days | 0.133 |
|  | *Fallopia japonica*, leaf 1, untreated, t=36 days | 0.130 |
| *Fallopia japonica*, leaf 2, paper, t=0 days | *Fallopia japonica*, leaf 2, paper, t=0 days | 2.845 |
|  | *Fallopia japonica*, leaf 2, paper, t=3 days | 2.352 |
|  | *Fallopia japonica*, leaf 2, paper, t=8 days | 1.878 |
|  | *Fallopia japonica*, leaf 2, paper, t=14 days | 2.423 |
|  | *Fallopia japonica*, leaf 2, paper, t=21 days | 2.245 |
|  | *Fallopia japonica*, leaf 2, paper, t=30 days | 2.186 |
|  | *Fallopia japonica*, leaf 2, paper, t=36 days | 2.148 |
| *Fallopia japonica*, leaf 2, 70% ethanol, t=0 days | *Fallopia japonica*, leaf 2, 70% ethanol, t=0 days | 2.845 |
|  | *Fallopia japonica*, leaf 2, 70% ethanol, t=3 days | 1.933 |
|  | *Fallopia japonica*, leaf 2, 70% ethanol, t=8 days | 1.583 |
|  | *Fallopia japonica*, leaf 2, 70% ethanol, t=14 days | 1.668 |
|  | *Fallopia japonica*, leaf 2, 70% ethanol, t=21 days | 1.183 |
|  | *Fallopia japonica*, leaf 2, 70% ethanol, t=30 days | 1.271 |
|  | *Fallopia japonica*, leaf 2, 70% ethanol, t=36 days | 1.496 |
| *Fallopia japonica*, leaf 2, untreated, t=0 days | *Fallopia japonica*, leaf 2, untreated, t=0 days | 2.845 |
|  | *Fallopia japonica*, leaf 2, untreated, t=3 days | 2.318 |
|  | *Fallopia japonica*, leaf 2, untreated, t=8 days | 2.221 |
|  | *Fallopia japonica*, leaf 2, untreated, t=14 days | 2.830 |
|  | *Fallopia japonica*, leaf 2, untreated, t=21 days | 1.697 |
|  | *Fallopia japonica*, leaf 2, untreated, t=30 days | 0.533 |
|  | *Fallopia japonica*, leaf 2, untreated, t=36 days | 0.294 |

**Table S2**

| Reference spectrum | Comparison spectrum | Bruker score |
| --- | --- | --- |
| *Impatiens glandulifera*, leaf 1, paper, t=0 days | *Impatiens glandulifera*, leaf 1, paper, t=0 days | 2.845 |
|  | *Impatiens glandulifera*, leaf 1, paper, t=3 days | 2.547 |
|  | *Impatiens glandulifera*, leaf 1, paper, t=8 days | 2.542 |
|  | *Impatiens glandulifera*, leaf 1, paper, t=14 days | 2.490 |
|  | *Impatiens glandulifera*, leaf 1, paper, t=21 days | 2.517 |
|  | *Impatiens glandulifera*, leaf 1, paper, t=30 days | 2.357 |
|  | *Impatiens glandulifera*, leaf 1, paper, t=36 days | 2.362 |
| *Impatiens glandulifera*, leaf 1, 70% ethanol, t=0 days | *Impatiens glandulifera*, leaf 1, 70% ethanol, t=0 days | 2.845 |
|  | *Impatiens glandulifera*, leaf 1, 70% ethanol, t=3 days | 2.184 |
|  | *Impatiens glandulifera*, leaf 1, 70% ethanol, t=8 days | 1.874 |
|  | *Impatiens glandulifera*, leaf 1, 70% ethanol, t=14 days | 1.951 |
|  | *Impatiens glandulifera*, leaf 1, 70% ethanol, t=21 days | 1.892 |
|  | *Impatiens glandulifera*, leaf 1, 70% ethanol, t=30 days | 1.796 |
|  | *Impatiens glandulifera*, leaf 1, 70% ethanol, t=36 days | 1.709 |
| *Impatiens glandulifera*, leaf 1, untreated, t=0 days | *Impatiens glandulifera*, leaf 1, untreated, t=0 days | 2.845 |
|  | *Impatiens glandulifera*, leaf 1, untreated, t=3 days | 2.497 |
|  | *Impatiens glandulifera*, leaf 1, untreated, t=8 days | 2.134 |
|  | *Impatiens glandulifera*, leaf 1, untreated, t=14 days | 1.527 |
|  | *Impatiens glandulifera*, leaf 1, untreated, t=21 days | 1.912 |
|  | *Impatiens glandulifera*, leaf 1, untreated, t=30 days | 0.960 |
|  | *Impatiens glandulifera*, leaf 1, untreated, t=36 days | 0.024 |
| *Impatiens glandulifera*, leaf 2, paper, t=0 days | *Impatiens glandulifera*, leaf 2, paper, t=0 days | 2.845 |
|  | *Impatiens glandulifera*, leaf 2, paper, t=3 days | 2.388 |
|  | *Impatiens glandulifera*, leaf 2, paper, t=8 days | 2.461 |
|  | *Impatiens glandulifera*, leaf 2, paper, t=14 days | 2.476 |
|  | *Impatiens glandulifera*, leaf 2, paper, t=21 days | 2.344 |
|  | *Impatiens glandulifera*, leaf 2, paper, t=30 days | 2.330 |
|  | *Impatiens glandulifera*, leaf 2, paper, t=36 days | 2.385 |
| *Impatiens glandulifera*, leaf 2, 70% ethanol, t=0 days | *Impatiens glandulifera*, leaf 2, 70% ethanol, t=0 days | 2.845 |
|  | *Impatiens glandulifera*, leaf 2, 70% ethanol, t=3 days | 2.154 |
|  | *Impatiens glandulifera*, leaf 2, 70% ethanol, t=8 days | 1.888 |
|  | *Impatiens glandulifera*, leaf 2, 70% ethanol, t=14 days | 1.988 |
|  | *Impatiens glandulifera*, leaf 2, 70% ethanol, t=21 days | 1.700 |
|  | *Impatiens glandulifera*, leaf 2, 70% ethanol, t=30 days | 1.790 |
|  | *Impatiens glandulifera*, leaf 2, 70% ethanol, t=36 days | 1.616 |
| *Impatiens glandulifera*, leaf 2, untreated, t=0 days | *Impatiens glandulifera*, leaf 2, untreated, t=0 days | 2.845 |
|  | *Impatiens glandulifera*, leaf 2, untreated, t=3 days | 2.414 |
|  | *Impatiens glandulifera*, leaf 2, untreated, t=8 days | 2.171 |
|  | *Impatiens glandulifera*, leaf 2, untreated, t=14 days | 1.006 |
|  | *Impatiens glandulifera*, leaf 2, untreated, t=21 days | 0.493 |
|  | *Impatiens glandulifera*, leaf 2, untreated, t=30 days | 0 |
|  | *Impatiens glandulifera*, leaf 2, untreated, t=36 days | 0 |

**Table S3**

| Reference spectrum | Comparison spectrum | Bruker score |
| --- | --- | --- |
| Untreated t=0 replicate 1 | Untreated t=0 replicate 2 | 2.528 |
|  | Untreated t=0 replicate 3 | 2.513 |
|  | Untreated -20°C replicate 1 | 2.329 |
|  | Untreated -20°C replicate 2 | 2.278 |
|  | Untreated -20°C replicate 3 | 2.265 |
|  | Untreated +5°C replicate 1 | 1.661 |
|  | Untreated +5°C replicate 2 | 2.163 |
|  | Untreated +5°C replicate 3 | 1.634 |
|  | Untreated +20°C replicate 1 | 0.713 |
|  | Untreated +20°C replicate 2 | 1.264 |
|  | Untreated +20°C replicate 3 | 0.421 |
|  | Untreated +30°C replicate 1 | 1.622 |
|  | Untreated +30°C replicate 2 | 1.231 |
|  | Untreated +30°C replicate 3 | 1.040 |
|  | Untreated +40°C replicate 1 | 0.992 |
|  | Untreated +40°C replicate 2 | 0.922 |
|  | Untreated +40°C replicate 3 | 0.813 |

**Table S4**

| Reference spectrum | Comparison spectrum | Bruker score |
| --- | --- | --- |
| Paper+70% ethanol t=0 replicate 1 | Paper+70% ethanol t=0 replicate 2 | 2.559 |
|  | Paper+70% ethanol t=0 replicate 3 | 2.520 |
|  | Paper+70% ethanol -20°C replicate 1 | 2.388 |
|  | Paper+70% ethanol -20°C replicate 2 | 2.346 |
|  | Paper+70% ethanol -20°C replicate 3 | 2.354 |
|  | Paper+70% ethanol +5°C replicate 1 | 2.518 |
|  | Paper+70% ethanol +5°C replicate 2 | 2.476 |
|  | Paper+70% ethanol +5°C replicate 3 | 2.521 |
|  | Paper+70% ethanol +20°C replicate 1 | 2.553 |
|  | Paper+70% ethanol +20°C replicate 2 | 2.102 |
|  | Paper+70% ethanol +20°C replicate 3 | 2.379 |
|  | Paper+70% ethanol +30°C replicate 1 | 2.507 |
|  | Paper+70% ethanol +30°C replicate 2 | 2.358 |
|  | Paper+70% ethanol +30°C replicate 3 | 2.439 |
|  | Paper+70% ethanol +40°C replicate 1 | 2.350 |
|  | Paper+70% ethanol +40°C replicate 2 | 2.439 |
|  | Paper+70% ethanol +40°C replicate 3 | 2.384 |

**Table S5**

| Reference spectrum | Comparison spectrum | Bruker score |
| --- | --- | --- |
| Paper t=0 replicate 1 | Paper t=0 replicate 2 | 2.642 |
|  | Paper t=0 replicate 3 | 2.539 |
|  | Paper -20°C replicate 1 | 2.247 |
|  | Paper -20°C replicate 2 | 2.111 |
|  | Paper -20°C replicate 3 | 2.345 |
|  | Paper +5°C replicate 1 | 2.503 |
|  | Paper +5°C replicate 2 | 2.289 |
|  | Paper +5°C replicate 3 | 2.313 |
|  | Paper +20°C replicate 1 | 2.374 |
|  | Paper +20°C replicate 2 | 2.472 |
|  | Paper +20°C replicate 3 | 2.323 |
|  | Paper +30°C replicate 1 | 2.427 |
|  | Paper +30°C replicate 2 | 2.519 |
|  | Paper +30°C replicate 3 | 2.440 |
|  | Paper +40°C replicate 1 | 2.529 |
|  | Paper +40°C replicate 2 | 2.384 |
|  | Paper +40°C replicate 3 | 2.377 |

**Table S6**

| Reference spectrum | Comparison spectrum | Bruker score |
| --- | --- | --- |
| Paper+70% isopropanol t=0 replicate 1 | Paper+70% isopropanol t=0 replicate 2 | 2.357 |
|  | Paper+70% isopropanol t=0 replicate 3 | 2.392 |
|  | Paper+70% isopropanol -20°C replicate 1 | 2.235 |
|  | Paper+70% isopropanol -20°C replicate 2 | 2.337 |
|  | Paper+70% isopropanol -20°C replicate 3 | 2.237 |
|  | Paper+70% isopropanol +5°C replicate 1 | 2.281 |
|  | Paper+70% isopropanol +5°C replicate 2 | 2.003 |
|  | Paper+70% isopropanol +5°C replicate 3 | 2.314 |
|  | Paper+70% isopropanol +20°C replicate 1 | 2.161 |
|  | Paper+70% isopropanol +20°C replicate 2 | 2.125 |
|  | Paper+70% isopropanol +20°C replicate 3 | 2.184 |
|  | Paper+70% isopropanol +30°C replicate 1 | 2.017 |
|  | Paper+70% isopropanol +30°C replicate 2 | 2.238 |
|  | Paper+70% isopropanol +30°C replicate 3 | 1.818 |
|  | Paper+70% isopropanol +40°C replicate 1 | 2.139 |
|  | Paper+70% isopropanol +40°C replicate 2 | 1.886 |
|  | Paper+70% isopropanol +40°C replicate 3 | 2.322 |
